# Strain Relaxation and Relative Defect Density with Thickness in MBE-Grown Ge_0.85_Sn_0.15_ on Ge(001)

**DOI:** 10.1021/acs.cgd.6c00174

**Published:** 2026-06-11

**Authors:** Dinesh Baral, Nirosh M. Eldose, Diandian Zhang, Hryhorii Stanchu, Fernando Maia de Oliveira, Serhii Kryvyi, Mohammad Zamani-Alavijeh, Mourad Benamara, Yuriy I. Mazur, Wei Du, Shui-Qing Yu, Gregory J. Salamo

**Affiliations:** a Institute for Nanoscience and Engineering, 3341 University of Arkansas, Fayetteville, Arkansas 72701, United States; b Department of Electrical Engineering and Computer Science, 3341 University of Arkansas, Fayetteville, Arkansas 72701, United States

## Abstract

Germanium–tin (GeSn) alloys are emerging as promising materials for mid-infrared optoelectronics and silicon-compatible photonic devices, owing to their tunable direct bandgap. However, the growth of high-quality GeSn films with high Sn content remains challenging due to strain-induced defect formation. In this study, we investigate the role of film thickness on strain-induced relaxation, defect density, and Sn segregation. A series of five samples with varying thicknesses and ∼15% Sn-containing GeSn layers were grown, ranging from the critical thickness for strain relaxation to the onset of Sn segregation. All GeSn samples were analyzed using X-ray diffraction reciprocal space mapping (XRD-RSM) to explore the evolution of strain-induced relaxation as a function of thickness. Photoluminescence measurements reveal that increasing the GeSn thickness enhances strain relaxation while reducing defect-related emission, indicating a decrease in effective defect density prior to reaching the threshold thickness of GeSn layer. At a thickness of ∼150 nm, the GeSn layer shows the onset of Sn segregation, evident in the XRD-RSM spectrum, marking the threshold thickness for Sn segregation. This work defines an effective growth window in terms of thickness (35 to 150 nm) for fabricating relaxed, defect-suppressed GeSn layers with 15% Sn content. These findings emphasize the crucial role of thickness control in balancing strain relaxation and defect suppression, advancing the fabrication of high-quality, high Sn-content relaxed GeSn using molecular beam epitaxy.

## Graphical abstract

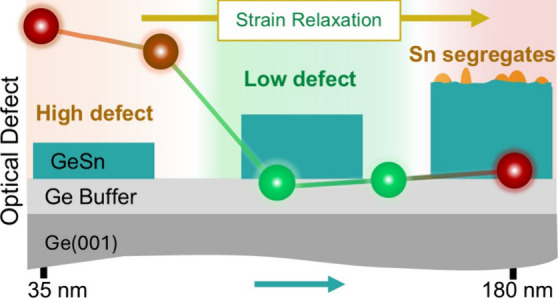

## Introduction

Germanium–tin (GeSn) alloys have emerged as a promising platform for infrared (IR) optoelectronics and silicon-compatible photonics, due to their tunable bandgap and potential to achieve a direct bandgap.^1–4^ Unlike pure Ge, which has an indirect bandgap, GeSn transitions from an indirect to a direct bandgap, when the Sn content exceeds 6%, depending on strain.^3–5^ For optimal mid-IR performance, a higher Sn content on the order of 15% is typically required to widen the offset between direct and indirect bandgap.^4,6–8^ However, growing high-quality GeSn films with high Sn content is challenging due to the low solid solubility of α-Sn in Ge (<1%) and the significant lattice mismatch (∼15%) between the Ge (5.6573 Å) and α-Sn (6.4892 Å).^9^ Although nonequilibrium growth can achieve high Sn content, the lattice mismatch results in strain, which induces relaxation creating defects and Sn segregation, all of which hinders the growth of high-quality GeSn films.

Chemical vapor deposition (CVD) has been the method of choice for growth of high-Sn content GeSn. The growth temperatures for CVD are at about 350 °C with high growth rates on the order of 10 nm/min. The growth strategy taken is to (i) grow on a Si substrate with a (ii) Ge buffer followed by (iii) GeSn growth until relaxation at a critical thickness, followed by (iv) further growth of GeSn to reduce defects.^10^ As a result, despite the large lattice mismatch, induced defects, and in some cases Sn segregation, CVD has produced sufficient quality GeSn samples to achieve high photoluminescence (PL) emission and the fabrication of IR lasers and detectors.^2,11,12^ However, CVD growth involves the thermal decomposition of precursors, which requires a high growth temperature and limits precise control over Sn incorporation in GeSn. In addition, CVD growth can limit the interfacial sharpness and makes it challenging to independently control elemental flux, growth rate, and thickness, as these growth parameters are strongly linked. In contrast, a less used method of growth for GeSn is molecular beam epitaxy (MBE), which is a flux-controlled growth method that offers precise control over the growth parameters such as composition, growth rate, growth temperature, and thickness down to the submonolayer level,^13,14^ is well suited for high-quality high-Sn GeSn growth of nanostructures and especially for high-quality interfaces for superlattice structures.^15–19^ However, MBE has a much lower growth rate and consequently requires a lower growth temperature to achieve a meaningful thickness of the GeSn film. Moreover, observing PL has also proved difficult from GeSn grown by MBE.^20–22^ For this reason, in this study, we investigate the growth process by MBE, especially examining relaxation, defect formation, and Sn segregation as a function of thickness of the GeSn layer with 15% Sn, grown on Ge(001) substrates, to establish a window of growth to achieve high-quality high-Sn content GeSn. We have chosen Ge substrates because they allow the growth of high-quality Ge buffer due to the lattice matching, which minimizes the defects and dislocations. This Ge buffer is then used to grow GeSn layers. This study will also serve as a model for the GeSn growth on Ge-buffered Si substrates. Investigating the defects in GeSn layers grown on Ge holds great value for exploring monolithic integration of GeSn with the Si platform along with the complementary metal oxide semiconductor (CMOS) compatibility.^23–26^

In this case, we used a similar strategy to CVD for our growth on (i) a Ge substrate with a (ii) Ge buffer followed by (iii) relaxation observed using X-ray diffraction reciprocal space mapping (XRD-RSM) and transmission electron microscopy (TEM) after a critical thickness of 35 nm, followed by (iv) further growth of GeSn to reduce defects observed by PL study. The observed critical thickness of 35 nm aligns well with the predictions from the People and Bean (P–B) model.^27,28^ According to the P–B model, when the areal strain energy density in the film exceeds the energy density required to form misfit dislocations at the film/substrate interface, dislocations will be generated to relieve the strain. The critical thickness of relaxation obtained using this P–B model has shown good agreement with experimental results across various Sn concentrations in GeSn films.^29^ Furthermore, we identified the threshold thickness of Sn segregation at 150 nm GeSn films containing 15% Sn. On the basis of these findings, we present a growth window, in terms of thickness, to achieve relaxed GeSn films with suppressed defects, from the critical thickness of relaxation to the onset of Sn segregation.

## Experimental Details

### Materials

GeSn layered structure with Sn content of ∼15% was grown on 2 in. Ge(001) wafers. The MBE system was equipped with Knudsen cells containing pyrolytic boron nitride (PBN) crucibles loaded with ultrahigh-purity (7N) intrinsic Ge and metallic Sn.

### Fabrication

All GeSn growths were done in an ultrahigh-vacuum (UHV) MBE chamber with a base pressure of 10^–11^ mbar. Prior to the growth, Ge wafers were preheated in the preparation chamber at 350 °C for 2 h followed by thermal degassing at 750 °C for 60 min to desorb the oxide layer. A 200 nm Ge buffer layer was first deposited at 450 °C to provide a clean and high-quality surface for the subsequent epitaxy. GeSn layers were then grown at a 140 °C for varying durations to obtain different film thicknesses, while keeping the Ge and Sn fluxes and growth temperature constant to ensure ∼15% Sn incorporation in all samples.

### Characterization

The sample growth process was monitored *in situ* using reflection high-energy electron diffraction (RHEED), which provided real-time information about the surface crystallinity. The surface morphology was investigated *ex situ* using the tapping mode atomic force microscopy (AFM), with a D3100 Nanoscope V scanning probe system (Bruker AXS, formerly Digital Instruments). The material structural, strain, and compositional characteristics were examined by using XRD with a Panalytical X’Pert Pro MRD, equipped with a 1.8 kW Cu Kα_1_ X-ray tube (λ = 1.540598 Å), a standard four-bounce Ge (220) monochromator, and a pixel detector. The surface morphology of the GeSn film, focusing on the migration of Sn segregates, was investigated with a Benchtop scanning electron microscope (SEM) from Electron Optics Instruments, equipped with a tungsten filament and an IXRF Systems EDS SDD X-ray Detector. Defect and strain evolution were investigated using an objective lens corrected TEM: a FEI Titan 80–300 microscope operating at 300 kV. TEM samples were prepared by mechanical polishing followed by ion milling. The PL studies were performed using a Horiba spectrometer coupled with a PbS detector.

## Results and Discussion

Figure 1 displays the structure of GeSn samples grown with different thicknesses to investigate strain relaxation as a function of thickness. Five samples were prepared with thicknesses of 35, 85, 138, 150, and 180 nm, labeled S1, S2, S3, S4, and S5, respectively. These samples were grown with constant flux of Sn and Ge to maintain the Sn content, but by varying the growth time to vary the film thickness. The growth temperature of 140 °C was observed to be sufficient to incorporate the supplied Sn content showing a smooth surface as evident from the AFM study. Figure 1 (bottom panel) shows AFM images of samples S1 to S5 with minimal root-mean-square (RMS) roughness indicating a change in roughness from S1 to S2 during relaxation and a second change in roughness from S3 to S4 during the onset of Sn segregation. The change from S4 to S5 is possibly due to the Sn segregation running across and smoothing the surface.

**Figure 1:**
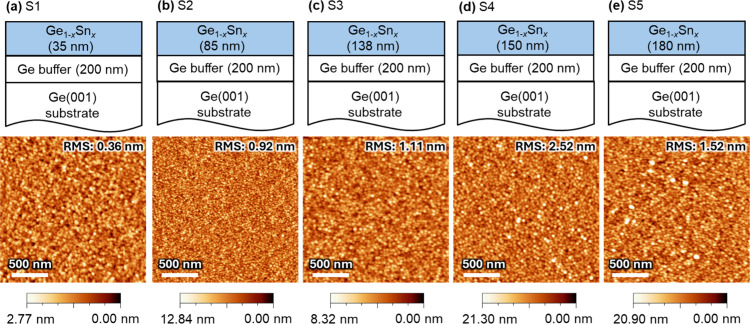
(a) S1, (b) S2, (c) S3, (d) S4, and (S5). Top panel: sample structure of GeSn with varying thicknesses for studying strain relaxation, grown on Ge(001). Bottom panel: corresponding AFM images of area 2 μm × 2 μm, showing the RMS roughness in the inset.

The crystal quality and the structural analysis of all five samples are investigated using HR-XRD. Figure 2 presents the strain relaxation behavior of the GeSn as a function of thickness. In particular, the 2θ/ω scans and RSMs for the (004) and (2̅2̅4) planes, respectively, depict the detailed structural characterization of the GeSn layers grown by MBE. For all samples, the GeSn peak appeared below the Ge peak due to the larger lattice spacing. The shape of the GeSn peak varies between samples due to the difference in degree of strain relaxation. For sample S1, the main GeSn peak is located on the *R* = 0% line, as shown in Figure 2a. A weak shoulder peak toward the relaxation line (*R* = 100%), showing a peak shift along the *Q*_*x*_, indicates the onset of strain relaxation and suggests a critical thickness of ∼35 nm, which is in close agreement with the P–B model^27,28^ and the experimentally observed critical thickness values for GeSn.^29–31^ With the increase of thickness above the critical value, the GeSn shoulder increases in intensity and shifts further toward the relaxation line, as can be seen on the RSMs of samples S2 to S5 (Figure 2b–e). For sample S2, the GeSn has grown with partial relaxation (∼35%) to the Ge substrate. Meanwhile, for S3, which has a thickness of 138 nm, significant relaxation (∼50%) has occurred without any evidence of Sn segregation. In contrast, a very weak low-Sn content GeSn peak is observed below the Ge substrate peak in sample S4 (Figure 2d), indicating the onset of Sn segregation at a thickness of 150 nm. In this case, further relaxation to 78% has occurred, and Sn segregation is detected. The Sn is lost from the top layers of the GeSn growth.

**Figure 2:**
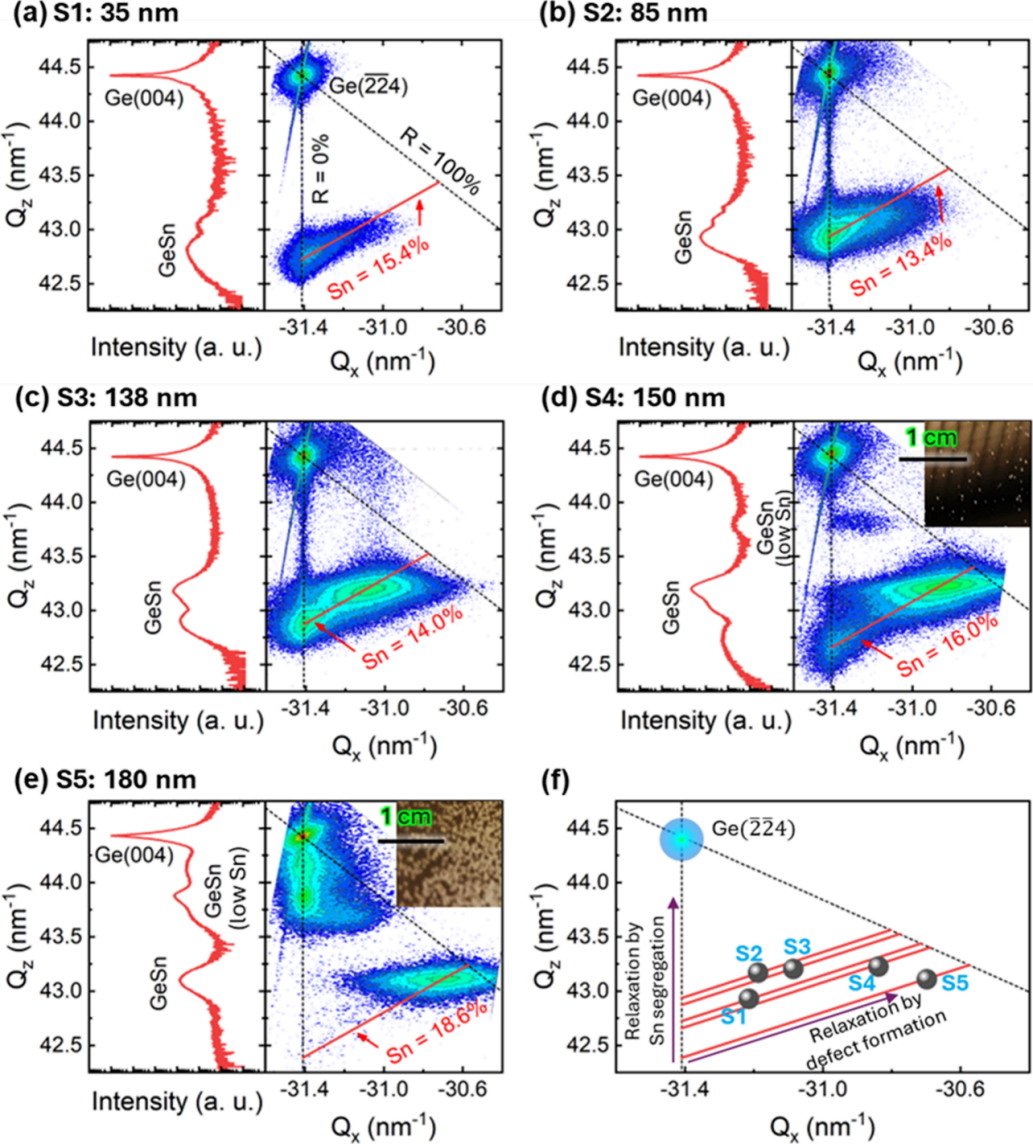
X-ray diffraction 2θ/ω profiles of symmetrical (004) reflection and RSMs of asymmetrical (2̅2̅4) reflection: (a–e) samples with increasing thickness and relaxation, from samples S1 through S5, with the GeSn thickness indicated, respectively. (f) Summary plot showing the mechanism, from the critical thickness of relaxation to the onset of Sn segregation. Insets: optical images showing surface morphology of sample S4 (in d) and of sample S5 (in e).

Likewise, the strong intensity of the low-Sn content peak in sample S5 (Figure 2e) indicates that a larger amount of Sn is segregated from a deeper layer into the GeSn growth. This is also evident in the optical image of the sample surface (inset of Figure 2e), which shows clear sign of Sn segregation. The appearance of the low-Sn GeSn peak in S4, which becomes more pronounced in S5, indicates the onset of Sn segregation, with a threshold thickness of approximately 150 nm (sample S4) for GeSn layers containing ∼15% Sn. From the analysis of the XRD-RSM for samples S1 to S5, it is observed that the strain relaxation occurs through two main mechanisms: (i) formation of defects in the film and (ii) Sn segregation on the surface, which helps relieve strain. These mechanisms are summarized in Figure 2f. We note that the GeSn film thickness was determined using a combination of characterization methods. For thinner samples S1 and S2 with pronounced Pendellösung fringes, thickness estimation was carried out using XRD, which provides good accuracy in this regime.^32^ With increasing thickness and strain relaxation, as in samples S3, S4, and S5, the Pendellösung fringes progressively weaken and eventually vanish, leading to high uncertainty in XRD-based thickness determination. Accordingly, the thickness of sample S3 was obtained directly from cross-sectional TEM study, as discussed in Figure 5. For samples S4 and S5, the GeSn layer thickness was estimated by extrapolating the growth time based on the thickness of S1, S2, and S3.

More insights about the strain relaxation during Sn segregation are revealed by SEM and XRD examination of sample S5. SEM imaging reveals two distinct surface regions, marked in Figure 3a,b as

(i) region A: areas of intact GeSn film with no Sn segregation, and
(ii) region B: areas are densely populated with Sn segregation.

**Figure 3:**
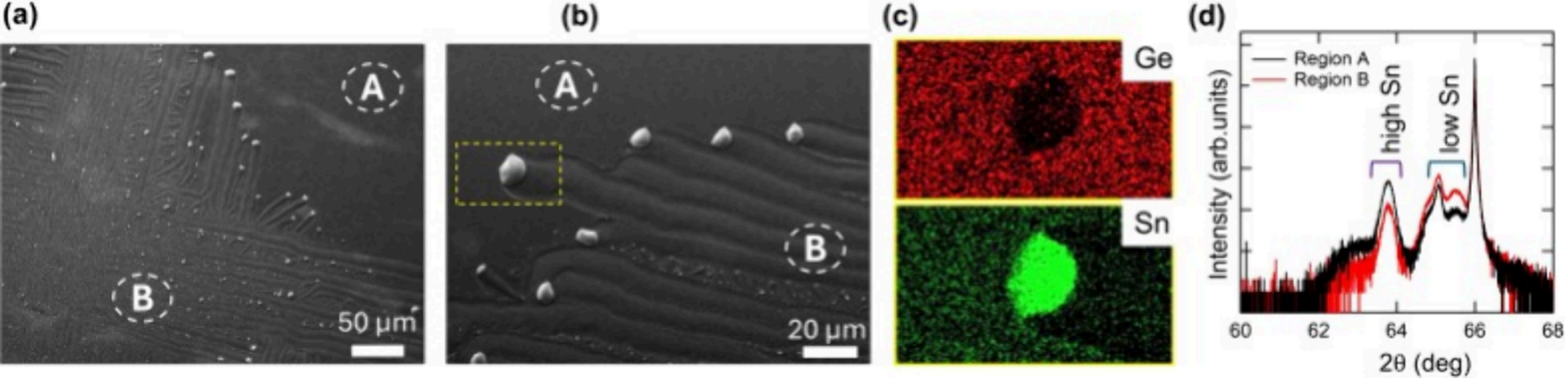
(a) SEM images showing the propagation of Sn droplets on the sample surface. (b) Zoomed-in view highlighting the migration of Sn droplets leading to GeSn film decomposition, region B. (c) SEM-EDX mapping of the region in (b) marked by the yellow dashed box. (d) XRD profiles corresponding to regions A and B.

Figure 3a reveals an agglomeration of Sn segregates (white dots and lines), where phase decomposition occurred. Under the optical microscope, such areas are distinguished as white spots on the sample surface. Moreover, multiple Sn segregates originate from the agglomeration and migrate along two orthogonal directions on the sample surface, further leading to film decomposition, as can be seen in Figure 3b. The white spots on the sample surface as observed in Figure 3b are confirmed to be Sn-rich segregates by SEM-EDX mapping, as shown in Figure 3c.

Moreover, two XRD measurements were performed by focusing the X-ray beam on the sample surfaces in areas A and B. The diffraction profiles, as shown in Figure 3c, reveal that the relative intensities of the low-Sn and high-Sn peaks depend on the surface coverage with Sn segregates. Specifically, the low-Sn peak is relatively stronger when measured from region B with a higher density of Sn agglomerations. This suggests that the high-Sn peak represents only the intact GeSn film, region A, that is not impacted by Sn droplet migration. Taken together, these SEM-EDX and spatially resolved XRD measurements support our interpretation that Sn segregation and droplet formation both contribute to the coexistence of strained low-Sn and relaxed high-Sn GeSn signals in the RSMs shown in Figure 2d,e.

In addition, for ease of comparison, Table 1 provides a summary of the GeSn layer thickness, Sn content, degree of strain relaxation, and RMS roughness for all five samples.

**Table 1:** Sample Properties: Thickness, Sn Composition, Roughness, and Degree of Strain Relaxation

samples	S1	S2	S3	S4	S5
thickness (nm)	35	85	138	150	180
Sn content (%)	15.0	13.4	14.0	16.0	18.0
RMS roughness (nm)	0.36	0.92	1.11	2.25	1.52
relaxation (%)	22	35	50	78	85

The GeSn layer growth rate and composition were also confirmed through SIMS for two selected samples, S1 and S3, as shown in Figure 4. These observed thickness values align with those observed from the XRD/TEM data. The growth rate, calculated from the data shown in Figure 4, indicates a linear relationship with time, validating the uniform growth rate of 0.35 nm/min, as the samples were grown under identical Ge–Sn flux and temperature conditions. At this low growth rate, sample growth requires several hours to reach the desired thickness. Nevertheless, the composition profiles of S1 and S3 show a uniform Sn distribution throughout each sample, with Sn contents of ∼15 and ∼14%, comparable to the XRD measurements. The uniform Sn distribution across the film thickness indicates that no significant growth time-dependent Sn redistribution is present, supporting the interpretation that strain relaxation is primarily governed by the film thickness.

**Figure 4:**
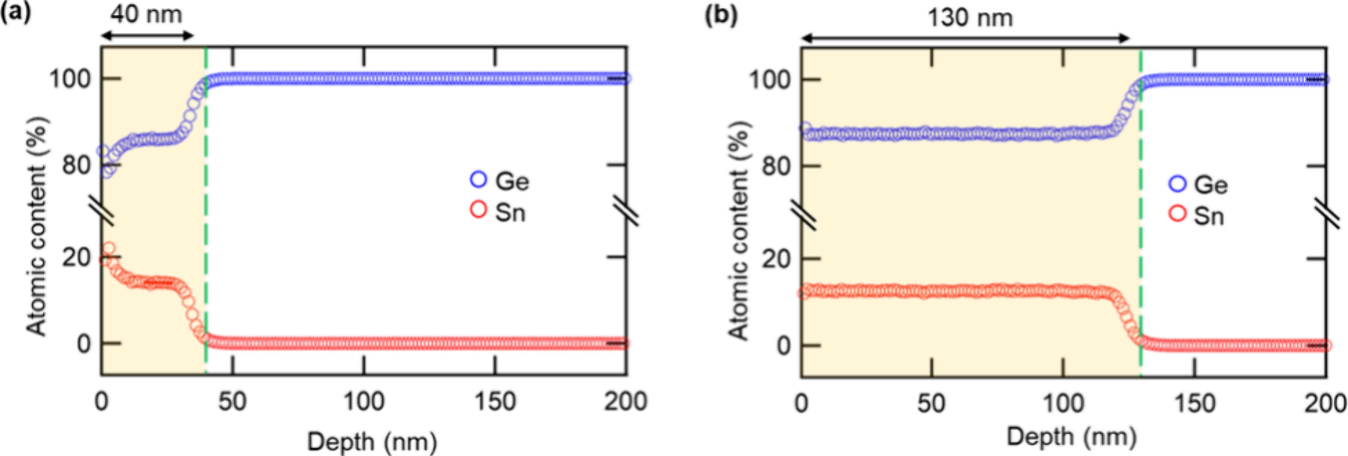
SIMS measurements of samples S1 in (a) and S3 in (b), illustrating thickness and the Sn distribution along the GeSn film grown on Ge(001).

To investigate the microstructural characteristics of strain relaxation along the growth direction, cross-sectional TEM (XTEM) was performed in sample S3, which has 50% relaxation and no sign of Sn segregation as seen in RSM (Figure 2c). Figure 5 presents the XTEM data on the evolution of strain relaxation and the crystal quality of the GeSn layer in sample S3. Low-magnification TEM analysis shown in Figure 5a uncovers a 220 nm Ge buffer layer grown on a Ge(001) substrate, followed by a 138 nm GeSn layer, closely matching the ∼130 nm thickness estimated by SIMS (Figure 4b). The GeSn layer shows a nonuniform distribution of defects, with distinct dark line patterns becoming apparent after the initial 35 nm of GeSn, marking the onset of the strain relaxation as shown in the high-magnification image in Figure 5b. This critical thickness (35 nm) for a GeSn layer with ∼15% Sn is consistent with the reported values^29–31^ and the observation from the XRD-RSM result (Figure 2a). To further investigate strain evolution, high-resolution TEM (HRTEM) images were analyzed using the fast Fourier transform (FFT). The FFT of the HRTEM (Figure 5c) reveals the spatial frequency components corresponding to the crystal’s lattice spacings, which appear as Bragg spots.^33^ The length variation of the reciprocal space vector ***g*** reflects the changes in the interplanar lattice spacing. Here, (002) and (2̅20) reflections were used to analyze in-plane and out-of-plane lattice variations, respectively. The HRTEM image was calibrated, assuming the Ge buffer on Ge(001) is fully relaxed. Analysis of the HRTEM data gives the following value of the reciprocal space vector ***g*** for three regions marked in Figure 5b as

(Region 1) Ge buffer: ***g***_(002)_ = 3.54 ± 0.02 nm^–1^ and ***g***_(220)_ = 5 ± 0.02 nm^–1^
(Region 2) Strained GeSn: ***g***_(002)_ = 3.45 ± 0.04 nm^–1^ and ***g***_(220)_ = 5 ± 0.02 nm^–1^
(Region 3) Partially relaxed GeSn: ***g***_(002)_ = 3.45 ± 0.04 nm^–1^ and ***g***_(220)_ = 4.88 ± 0.02 nm^–1^

**Figure 5:**
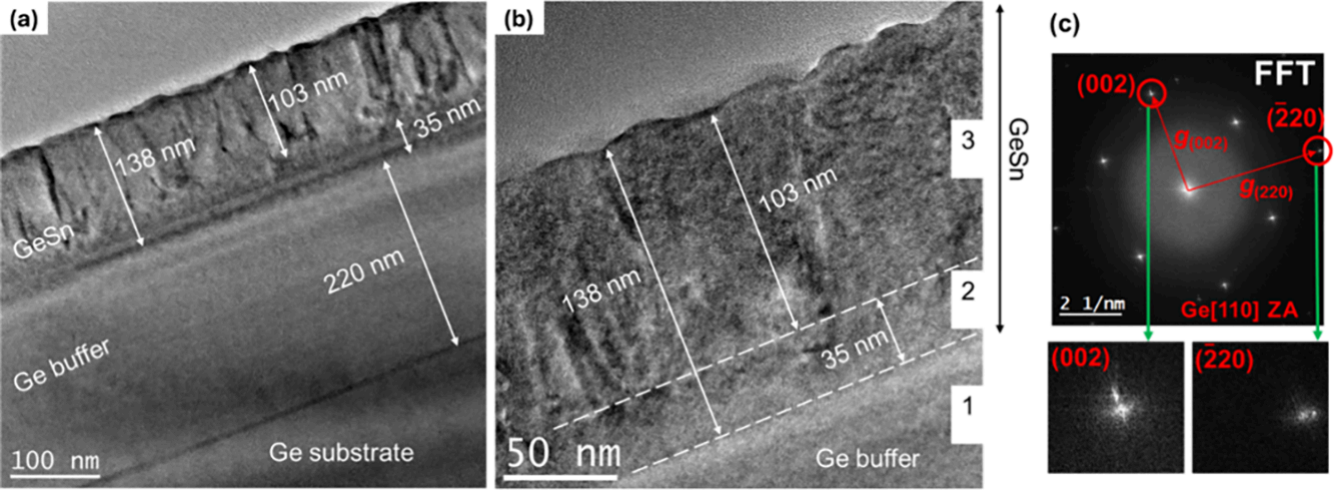
XTEM study of sample S3. (a) Low-magnification image showing the sample structure GeSn/Ge buffer/Ge(001). (b) HRTEM image highlighting the transition from strained (area between the dashed white lines) to relaxed regions in the GeSn layer. (c) FFT of the HRTEM image in (b) revealing the Bragg spots corresponding to lattice spacing.

These values show that the in-plane lattice spacing of the first 35 nm of the GeSn layer matches that of the Ge buffer, indicating that the first 35 nm of the GeSn layer remains fully strained. Beyond 35 nm, the GeSn layer begins to relax, as indicated by a decrease in the length of the ***g***_(220)_ vector and supplemented by the increase in relaxation. This result is in good agreement with the XRD data (Figure 2c), which shows the presence of both fully strained and partially relaxed layers.

To investigate the optical quality of the samples and explore the evolution of defects related to the strain relaxation and GeSn layer thickness, a detailed PL study was performed. Figure 5 shows the PL spectra of samples S1 to S5 at 10 K using laser excitation of 532 nm. The shorter penetration depth of the laser 532 nm into the GeSn/Ge structure allowed the laser to probe only the top ∼20 nm of the GeSn film;^34^ therefore, the Ge emission that originates from the buffer/substrate is absent, as shown in Figure 6a–f. In these spectra, only a defect emission around 2420 nm is observed for all samples. The PL emission at 2420 nm represents the recombination of carriers mediated through a defect state within the bandgap that acts as a trap for carriers relaxing down from the conduction band to the valence band.^35,36^ Here, changes in defect density are evaluated relatively from the thinner sample S1 to thicker sample S5 using the defect-mediated PL intensity, providing a comparative measure of the defect evolution with thickness and strain relaxation. The attribution of the 2420 nm signal observed in the photoluminescence presented in Figure 6a–f of the manuscript to a defect emission is based first on previous reports on the defect emission in Ge and GeSn structures, and ultimately in our own recent studies. As an example of the existing literature on the defect emission in GeSn materials, Rogowicz et al.^37^ observed the defect emission in GeSn films with different Sn contents at 20 K, matching the same energy of the Ge substrate and being independent of Sn content. Our group recently evidenced this same emission in a graded GeSn film^21^ and on GeSn/Ge superlattices^38^ at temperatures below 150 K. The early studies on the origin of this emission were done investigating pure Ge crystals with different degrees of dislocation densities. In particular, Barth et al.^39^ observed that Ge crystals with high density of 60° dislocations exhibited an intense luminescent emission at the energy of ∼0.51 eV at 14 K, which represents about 2430 nm in wavelength. Also, Izotov et al.^40^ showed that this emission, named “dislocation photoluminescence” of Ge, was affected by plastic deformation in Ge crystals. Recently, Gupta et al.^41^ observed 60° dislocations propagating from Ge substrate into a GeSn film and creating donor-like defect states evidenced by deep-level transient spectroscopy. The defect emission also prevents the observation of the direct and indirect bandgap transitions of the GeSn/Ge structure, and the corresponding diagram of energy levels is shown in Figure 6h. Figure 6g summarizes the PL intensity from defects for all the samples and compares to the Ge buffer (reference). The variation in PL intensity from defects reflects the changes in the defect density of the GeSn film. In sample S1, the defect peak is stronger compared to the Ge reference, as the sample begins to relax, suggesting the development of defects. As growth progresses from samples S1 to S2, the PL intensity from defects decreases slightly, indicating a reduction in defect density. With further thickness increase in S3 (∼138 nm), defects are significantly reduced, improving the film quality with no signs of Sn segregation. However, when the thickness reaches ∼150 nm (sample S4), the onset of Sn segregation is observed, indicating strain relaxation through segregation. Beyond this point, further increase in thickness leads to more pronounced Sn segregation, as discussed above and presented in Figures 2e and 3 for sample S5. In transition from S4 to S5, an increase in defect density is observed, correlating with both the Sn segregation and higher relaxation.

**Figure 6:**
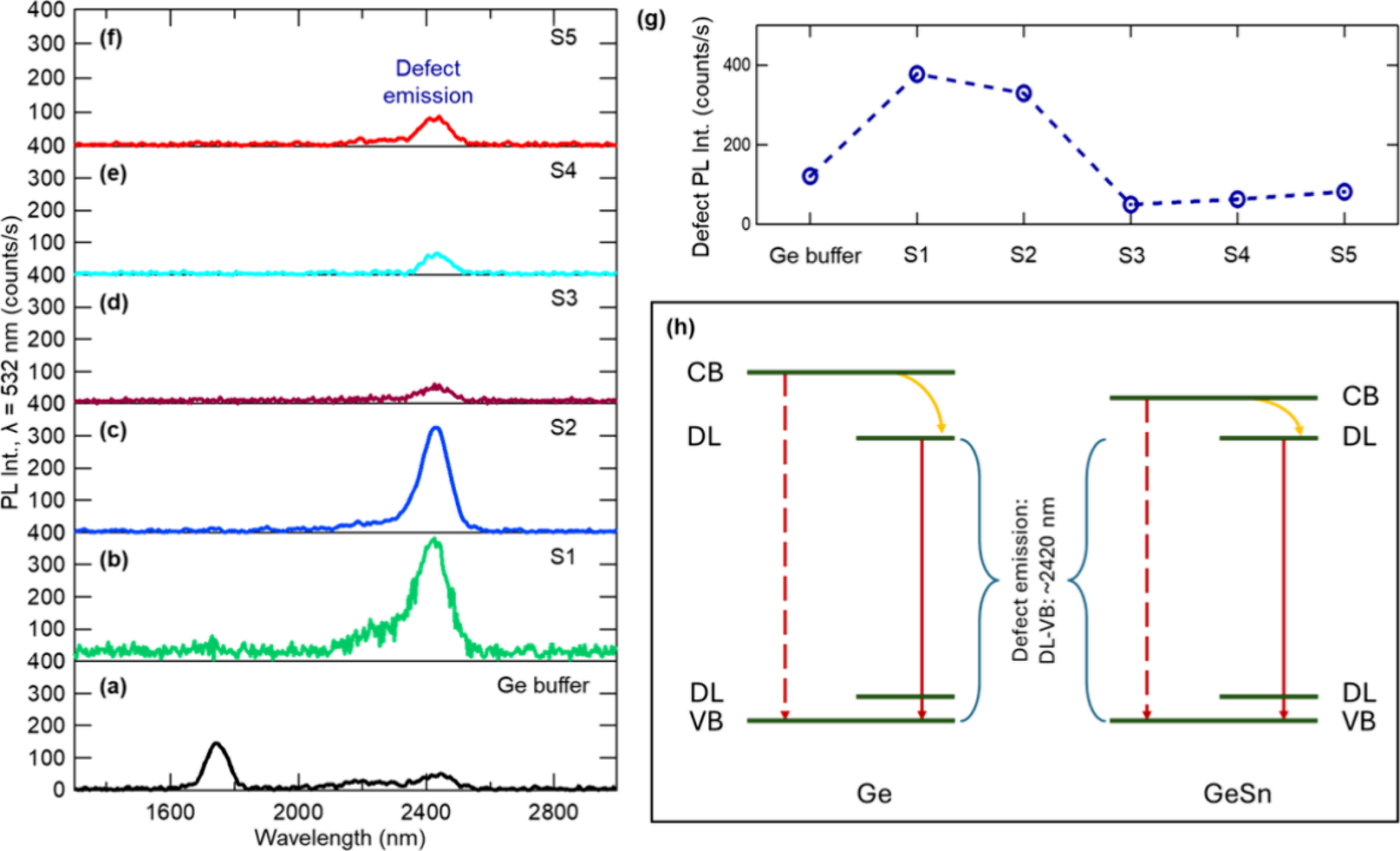
PL measurement of (a) Ge buffer (reference), (b) S1, (c) S2, (d) S3, (e) S4, and (f) S5 using laser of 532 nm at 10 K. (g) Summarized plot showing the evolution of PL intensity of the defect emission for sample shown in (a–f). (h) Diagram of energy levels illustrating transitions from conduction band (CB) to valence band (VB) and mediated through defect level (DL).

## Conclusions

This study explores the strain relaxation, relative defect density, and Sn segregation behavior in GeSn with ∼15% Sn content grown on Ge(001) by MBE at 140 °C. A series of five GeSn samples with thicknesses ranging from 35 to 180 nm were analyzed, revealing a critical thickness for the onset of strain relaxation at ∼35 nm, consistent with the P–B model. At 35 nm, the GeSn layer is ∼22% relaxed with relaxation induced defects. With further growth to 85 nm, the defect density slightly drops, and at 138 nm, the defect density drops significantly despite the increase in relaxation (35 to 50%) due to the filtering. Similarly, a further small increase in thickness from 138 to 150 nm significantly raises relaxation (50 to 78%), which correlates with the slightly higher defect densities and the onset of Sn segregation, indicating a threshold thickness for Sn segregation ∼150 nm in GeSn with 15% Sn. Notably, Sn segregation becomes obvious in the 180 nm-thick sample. These results define the growth thickness for high-quality GeSn/Ge(001) with 15% Sn content grown by using MBE, indicating that a GeSn thickness of about 138 nm on Ge is a favorable choice.

## Data Availability

The data are available from the corresponding author upon reasonable request.
